# Supplementary material for: MetaRibo-Seq measures translation in microbiomes
Source: Nat Commun. 2020 Jun 29;11:3268. doi: 10.1038/s41467-020-17081-z (PMC7324362; doi:10.1038/s41467-020-17081-z)
Supplement: Supplementary file 10 — Supplementary Data 7 [file 41467_2020_17081_MOESM10_ESM.zip › File2/Confidence_High_Taxonomy/83466_out.krona.html]

Javascript must be enabled to view this page.

members
magnitude
magnitudeUnassigned
count
unassigned
taxon
rank

83466\_out

133

133
superkingdom
2

1239
phylum
133

class
186801
133

186802
order
133


SRS045244\_contig\_number\_contig-100\_20523.20524
133
186803
family
1

132
841
genus

species
166486

SRS142503\_contig\_number\_3663
1

species
1263105

SRS077194\_contig\_number\_10629
1

species
1262941

SRS014613\_contig\_number\_20000SRS015579\_contig\_number\_1738SRS016495\_contig\_number\_2128SRS019381\_contig\_number\_20846SRS020233\_contig\_number\_contig-100\_2260.225051SRS023346\_contig\_number\_contig-100\_21349.21349SRS042628\_contig\_number\_24548SRS043667\_contig\_number\_contig-100\_906.36435SRS043841\_contig\_number\_3170SRS044535\_contig\_number\_19617SRS048060\_contig\_number\_8394SRS050941\_contig\_number\_2378SRS056273\_contig\_number\_1778SRS058723\_contig\_number\_1086SRS063040\_contig\_number\_24946SRS075821\_contig\_number\_15123SRS076804\_contig\_number\_17967SRS077294\_contig\_number\_contig-100\_20515.20515SRS077335\_contig\_number\_9031SRS097889\_contig\_number\_27450SRS100907\_contig\_number\_contig-100\_31578.31579SRS104311\_contig\_number\_5536SRS105153\_contig\_number\_7526SRS1055034\_contig\_number\_9617SRS140492\_contig\_number\_30715SRS143148\_contig\_number\_18669SRS143466\_contig\_number\_contig-100\_16956.69624SRS143780\_contig\_number\_3246SRS146813\_contig\_number\_21952SRS147346\_contig\_number\_31932SRS149879\_contig\_number\_12979SRS893341\_contig\_number\_2704
32


SRS014923\_contig\_number\_41567SRS015854\_contig\_number\_1119SRS017859\_contig\_number\_7240SRS020869\_contig\_number\_30116SRS023526\_contig\_number\_30017SRS024132\_contig\_number\_23945SRS047014\_contig\_number\_20565SRS075716\_contig\_number\_contig-100\_395.35451SRS1041136\_contig\_number\_18451SRS146812\_contig\_number\_27183SRS148253\_contig\_number\_9793SRS148424\_contig\_number\_19325SRS893383\_contig\_number\_31306
13
species
2293144

species
1897017

SRS011302\_contig\_number\_18980SRS013638\_contig\_number\_contig-100\_3453.74739SRS013687\_contig\_number\_20309SRS014235\_contig\_number\_7097SRS015431\_contig\_number\_47682SRS015578\_contig\_number\_14647SRS019397\_contig\_number\_1809SRS021484\_contig\_number\_4615SRS050925\_contig\_number\_26914SRS063518\_contig\_number\_15425SRS098571\_contig\_number\_1988SRS098827\_contig\_number\_contig-100\_2168.75873SRS147377\_contig\_number\_contig-100\_9851.51304SRS148091\_contig\_number\_7338
14


SRS017821\_contig\_number\_4905SRS051882\_contig\_number\_20318SRS077641\_contig\_number\_5235SRS147919\_contig\_number\_17836
4
species
301302

species
2049040
67

SRS012273\_contig\_number\_46447SRS012969\_contig\_number\_13552SRS013951\_contig\_number\_17900SRS014313\_contig\_number\_4782SRS014412\_contig\_number\_14912SRS014459\_contig\_number\_contig-100\_228.136490SRS014979\_contig\_number\_29841SRS015065\_contig\_number\_28582SRS015663\_contig\_number\_12201SRS016132\_contig\_number\_contig-100\_5731.5732SRS016989\_contig\_number\_15510SRS017433\_contig\_number\_6383SRS018817\_contig\_number\_19341SRS019068\_contig\_number\_37692SRS019161\_contig\_number\_6024SRS019445\_contig\_number\_contig-100\_497.171684SRS019787\_contig\_number\_11373SRS021948\_contig\_number\_34275SRS024435\_contig\_number\_contig-100\_21627.103611SRS046502\_contig\_number\_10610SRS046712\_contig\_number\_2077SRS050422\_contig\_number\_26427SRS051031\_contig\_number\_2696SRS052027\_contig\_number\_4566SRS053214\_contig\_number\_19108SRS053398\_contig\_number\_contig-100\_27207.66082SRS054352\_contig\_number\_contig-100\_35422.35422SRS054905\_contig\_number\_contig-100\_29257.29258SRS054956\_contig\_number\_11411SRS055017\_contig\_number\_28610SRS058070\_contig\_number\_25705SRS058770\_contig\_number\_contig-100\_17427.17427SRS063127\_contig\_number\_362SRS064557\_contig\_number\_3959SRS064645\_contig\_number\_contig-100\_68.68SRS065504\_contig\_number\_27596SRS075021\_contig\_number\_136SRS075078\_contig\_number\_18873SRS075341\_contig\_number\_contig-100\_6940.90033SRS075878\_contig\_number\_3892SRS075984\_contig\_number\_contig-100\_28370.28371SRS076756\_contig\_number\_14126SRS077024\_contig\_number\_contig-100\_13623.59332SRS077231\_contig\_number\_17668SRS077730\_contig\_number\_12716SRS078242\_contig\_number\_11690SRS078419\_contig\_number\_30644SRS097920\_contig\_number\_22738SRS098881\_contig\_number\_5505SRS100021\_contig\_number\_10816SRS101433\_contig\_number\_10848SRS1041036\_contig\_number\_5444SRS104485\_contig\_number\_503SRS1055022\_contig\_number\_10427SRS1055049\_contig\_number\_10637SRS1055056\_contig\_number\_9436SRS1055076\_contig\_number\_contig-100\_782.95195SRS1055083\_contig\_number\_2552SRS142542\_contig\_number\_25060SRS143417\_contig\_number\_19886SRS143722\_contig\_number\_12276SRS144135\_contig\_number\_28482SRS144183\_contig\_number\_29403SRS144297\_contig\_number\_7409SRS148159\_contig\_number\_31030SRS148721\_contig\_number\_15958SRS149784\_contig\_number\_4735
